# Fall Course of Lectures on Anatomy and Surgery

**Published:** 1842-08

**Authors:** 


### FALL COURSE OF LECTURES ON ANATOMY AND OPERATIVE SURGERY.

We would remind our friends abroad, that Professors Cobb and Gross will deliver a course of lectures on the above branches of Medical Science, in the Louisville Medical Institute, commencing the 21st of September, and terminating on the last Saturday of October. It would be needless to set forth the benefit to be derived from these lectures, which will embrace visceral and surgical anatomy, with operations on the dead subject. Too little attention is paid to these subjects, more especially to operative surgery, and yet they are as indispensable to one that aspires to be an accomplished surgeon, as are *a*, *b*, *c* and *syllabication* to one that wishes to make a scholar. We hope the young men of the west will avail themselves of this opportunity, and be in early attendance on these lectures. C.

